# Neural correlates of cognitive intervention in persons at risk of developing Alzheimer’s disease

**DOI:** 10.3389/fnagi.2014.00231

**Published:** 2014-08-26

**Authors:** S. M. Hadi Hosseini, Joel H. Kramer, Shelli R. Kesler

**Affiliations:** ^1^Department of Psychiatry and Behavioral Sciences, Stanford University School of MedicineStanford, CA, USA; ^2^Department of Neurology, Memory and Aging Center, University of CaliforniaSan Francisco, CA, USA; ^3^Stanford Cancer InstitutePalo Alto, CA, USA

**Keywords:** cognitive training, mild cognitive impairment (MCI), aging, plasticity, neuroimaging, cognitive stimulation, computerized training

## Abstract

Cognitive training is an emergent approach that has begun to receive increased attention in recent years as a non-pharmacological, cost-effective intervention for Alzheimer’s disease (AD). There has been increasing behavioral evidence regarding training-related improvement in cognitive performance in early stages of AD. Although these studies provide important insight about the efficacy of cognitive training, neuroimaging studies are crucial to pinpoint changes in brain structure and function associated with training and to examine their overlap with pathology in AD. In this study, we reviewed the existing neuroimaging studies on cognitive training in persons at risk of developing AD to provide an overview of the overlap between neural networks rehabilitated by the current training methods and those affected in AD. The data suggest a consistent training-related increase in brain activity in medial temporal, prefrontal, and posterior default mode networks, as well as increase in gray matter structure in frontoparietal and entorhinal regions. This pattern differs from the observed pattern in healthy older adults that shows a combination of increased and decreased activity in response to training. Detailed investigation of the data suggests that training in persons at risk of developing AD mainly improves compensatory mechanisms and partly restores the affected functions. While current neuroimaging studies are quite helpful in identifying the mechanisms underlying cognitive training, the data calls for future multi-modal neuroimaging studies with focus on multi-domain cognitive training, network level connectivity, and individual differences in response to training.

## Introduction

Alzheimer’s disease (AD) is the most common form of dementia, a general term for memory loss and other intellectual abilities serious enough to interfere with daily life. Recent World Alzheimer’s Report indicates that over 35 million people worldwide have AD in 2013 and this number is expected to triple by 2050 (Prince et al., 2013). Alzheimer’s is not solely a cognitive problem. In the United States, AD is the sixth leading cause of death across all ages (Murphy et al., 2013) and over 500,000 deaths annually may be attributable to AD in older adults aged 75 years and older in 2010 (James et al., 2014). While it has been more than 100 years since AD was first identified, there are still no effective disease-modifying drugs available for this (Buschert et al., 2010). The number of mortalities attributed to AD is still increasing while the number of deaths attributed to other fatal diseases (heart disease, cancer, and stroke) has decreased (Murphy et al., 2013), calling for development of new cost-effective treatments.

Cognitive training is a guided practice on a set of standard tasks designed to increase particular cognitive functions that further supports accomplishments of everyday tasks and independent living (Lindesay et al., 2010; Rebok et al., 2014). It has been shown that cognitive training promotes several neuroplastic mechanisms in the brain that can be conserved well into advanced age (Nyberg et al., 2003; Boyke et al., 2008; de Villers-Sidani et al., 2010). Candidate cellular mechanisms underlying gray matter plasticity include axon sprouting, dendritic branching and synaptogenesis, neurogenesis and glial changes whereas the mechanisms underlying white matter changes include myelination, changes in fiber organization, astrocyte changes and angiogenesis (Zatorre et al., 2012). Thus, cognitive training has begun to receive increased attention in recent years as a non-pharmacological, cost-effective intervention and treatment of AD.

There has been increasing behavioral evidence demonstrating training-related improvement in cognitive performance in early stages of AD (Cipriani et al., 2006; Barnes et al., 2009; Kinsella et al., 2009; Li et al., 2011; Gagnon and Belleville, 2012; Herrera et al., 2012; Moro et al., 2012; Rovner et al., 2012; Valdes et al., 2012; Gaitán et al., 2013; Greenaway et al., 2013; Reijnders et al., 2013). While these studies provide important insight about the efficacy of cognitive training, neuroimaging studies are crucial to pinpoint changes in brain structure and function associated with cognitive training. Previous reviews on neuropathology of AD suggest distributed changes in brain structure and function comprising temporal, frontoparietal, and default mode networks (Buckner et al., 2005; Bokde et al., 2009; Seeley et al., 2009; Browndyke et al., 2013; Radanovic et al., 2013). These widespread neuronal changes suggest the importance of neuroimaging studies to investigate the overlap between neural networks rehabilitated by the training procedure and those affected in AD.

Moreover, multi-modal neuroimaging studies can further our understanding of neuroanatomical and functional mechanisms underlying cognitive training. Specifically, cognitive training can result in improved performance in various ways: training might result in rehabilitation (or normalization) of the affected structure (or function) and/or reorganization of alternate networks to compensate for the role of the affected regions (Kelly et al., 2006a). Training programs must ideally favor the rehabilitation/normalization mechanisms and ensure that the compensatory mechanisms do not negatively impact the intact functions through sharing resources (Behrmann et al., 2005). Understanding the training-related mechanisms is crucial to design efficient and effective cognitive training programs for treatment/intervention of AD.

In this review article, we provide an overview of the functional and structural neuroimaging studies on cognitive training in normal aging and persons at risk of developing AD to identify the potential mechanisms underlying current cognitive training procedures. We will then discuss the limitations of the current studies and address the implications of the findings for future research.

## Functional and structural plasticity in old age

Recent findings suggest that cognitive training results in neuroanatomical and functional changes that extend into advanced age. Nyberg et al. (2003) were among the first to show changes in occipito-parietal activity in older adults who benefited from memory training using O-15 H_2_O PET. Since then, several functional MRI and PET studies have shown training-related changes in brain activity in healthy older adults [see Belleville and Bherer, 2012 for a review]. It has been shown that cognitive training can reduce age differences in ventral and dorsal prefrontal activation (Erickson et al., 2007) and decrease neocortical brain activity observed with functional MRI (Brehmer et al., 2011), and increase resting cerebral blood flow to the default-mode network and central executive network observed with perfusion MRI (Mozolic et al., 2010; Chapman et al., 2013) in older adults. These findings provide evidence for functional plasticity in old age and suggest a mixed pattern of increased and decreased activation in response to training in healthy older adults.

Evidence regarding training-related structural plasticity in old age is more recent. Boyke et al.’s (2008) study was one of the earliest successful attempts that showed increased gray-matter volume in the middle temporal regions in older adults after 3 months of training on a three-ball cascade juggling. Recent studies have repeatedly demonstrated structural plasticity associated with cognitive training in healthy older adults. Cognitive training resulted in increased thickness in the right insula, left lateral orbitofrontal and fusiform cortices (Engvig et al., 2010), stabilized hippocampal volume (Lövdén et al., 2012), increased fractional anisotropy (FA) and decreased mean diffusivity (MD) in the genu of corpus callosum (Lövdén et al., 2010; Engvig et al., 2012a), and increased FA in the frontal white matter tracts (Engvig et al., 2012a) in response to training. These data support experience-dependent plasticity of gray and white matter structure in advanced age. Unlike the mixed pattern of changes in functional plasticity, structural changes consistently show an increased pattern (or reduced age-related decline). The above functional and structural neuroimaging evidence suggest that cognitive training can be employed to restore neuroanatomical decline associated with aging.

## Pattern of training-related changes in brain function and structure in persons at risk of AD

**Figure 1:**
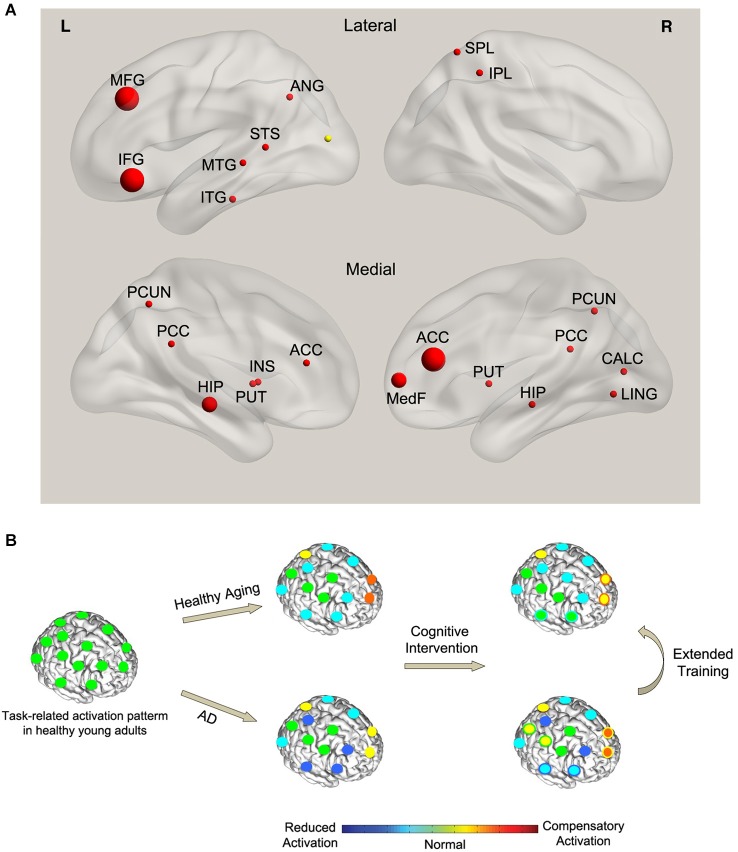
**(A)** Effect of cognitive training on functional brain activity in AD. The spheres indicate the regions that showed significant training-related changes in AD compared with controls. The size of the spheres corresponds to the frequency of observed activations across studies. Red (yellow) color indicates training-related increase (decrease) in activity. **(B)** A conceptual model of the effect of training on task-related functional activity in AD. Left panel: the normal pattern of brain activity during a hypothetical task in healthy young adults (green circles). Middle panel: with normal aging, task performance is associated with a combination of reduced activity in task-specific brain regions (cyan circles) and compensatory activation in the alternate networks (orange and yellow circles). However, compared with healthy aging, AD results in a more pronounced reduction in the activity of some of the task-specific regions (blue circles) and a breakdown of the compensatory network (yellow circles). Right panel: cognitive training in healthy aging leads to normalization of brain activity in some of the task-specific regions (green circles with cyan boundary) as well as reduced compensatory activations (yellow circles with orange boundary). This pattern is consistent with the cognitive training literature showing a combination of training-related increase (normalization) and decrease (less compensation) in activity in healthy aging. On the other hand, training in AD leads to partly restoration of the activity in some of the task-specific regions (cyan circles with blue boundary) parallel with recovery of part of the compensatory network (orange circles with yellow boundary) and recruitment of new compensatory networks (yellow circles with green boundary). This pattern is consistent with the observed dominant increase in brain activity (combination of restoration and compensation) in AD after training.

There have been a few efforts to examine changes in brain function and structure in response to cognitive training in AD. Most of these studies focus on older adults with mild cognitive impairment (MCI), a risk factor for developing dementia. We summarized the results of functional MRI studies that investigated the effect of cognitive training in persons at risk of developing AD (Table 1). We searched PubMed for the keywords “Alzheimer’s disease” or “MCI” or “Mild Cognitive Impairment”, and “Cognitive Intervention” or “Cognitive Training” or “Cognitive Stimulation”, and “functional MRI” or “fMRI” or “PET” or “Positron Emission Tomography” or “EEG” or “Electroencephalography” or “MEG” or “Magnetoencephalography” or “Brain”. Only studies that examined the effect of training on brain function using functional MRI were included in the summary, since we could not aggregate the results of EEG/MEG and PET studies with fMRI. However, the corresponding results were briefly noted. We did not include case studies in the analysis (Clare et al., 2009; van Paasschen et al., 2009). Non-English, non-human studies were not included. Five studies fulfilled the inclusion criteria (see Table 1 for details). No additional studies were found using other databases (e.g., Web of Science) and search engines (e.g., Google Scholar). Since most of the studies did not report the coordinates of observed changes in brain activity, we could not perform a systematic meta-analysis (e.g., activation likelihood estimation, ALE; Eickhoff et al., 2009). Instead, we assigned the reported regions (or coordinates when they are available) to corresponding region of interest (ROI) in Automated Anatomical Labeling (AAL) atlas (Tzourio-Mazoyer et al., 2002) and summarized the findings based on the frequency of the observed activations in each ROI across studies (Figure 1A). The observed pattern suggests a consistent increase (or attenuated decline) in activity of distributed brain regions, including hippocampal, prefrontal, and posterior default mode network in response to training. These results are corroborated by PET and EEG studies that reported increased metabolic activity and functional plasticity after training in AD (Forster et al., 2011; Spironelli et al., 2013). It should be noted that the observed pattern of activity is confounded by different types of training programs and outcomes involved in the studies and thus makes it difficult to compare the effect between clinical groups.

**Table 1:** **The list of the included functional MRI studies**.

Study	Cognitive training	Target	Control	Duration	fMRI task
Belleville et al. (2001)	Memory training	MCI	HC-active	6 w	Face-name assoc. (memory)
Carlson et al. (2009)	Experience corps	MCI	MCI-waitlist	6 m	Flanker (executive function)
Hampstead et al. (2011)	Memory training	MCI	MCI*	2 w	Face-name assoc. (memory)
Hampstead et al. (2012)	Memory training	MCI	HC-active	2 w	Face-name assoc. (memory)
Rosen et al. (2011)	Auditory processing	MCI	MCI-active	2 m	Auditory-verbal (memory)

w: weeks; m: months; * within-subjects control condition.

Regarding the underlying mechanism, the above studies reported both functional normalization and compensation in response to training. Specifically, restoration of hippocampal activity was reported in several memory-training studies (Rosen et al., 2011; Hampstead et al., 2012). The restoration of activity in task-specific regions was parallel with increased activity in alternative brain regions that were not active at baseline in comparison with controls (Belleville et al., 2011; Hampstead et al., 2011, 2012) that suggests compensatory recruitment of alternative networks.

The dominant training-related increase in functional activity in persons at risk of developing AD (Figure 1A) differs from the observed pattern in healthy aging which shows a combination of increase and decrease in activity. It has been shown that changes in brain function antedate the cognitive decline and pathological effects of amyloid deposition in persons at risk of developing AD (Sheline et al., 2010). MCI has been associated with prominent changes in brain function [see Browndyke et al., 2013; Risacher and Saykin, 2013 for a review]. We speculate that the dominant training-related increase in brain activity in MCI subjects signals mainly compensation and partly normalization of the affected functions. On the other hand, in healthy aging, training results in a decrease in the recruitment of compensatory regions (decrease in activity) as well as an increase in brain activity which indicates functional normalization of the regions affected by normal age-related decline. The latter is supported by the data showing an increase in hemispheric asymmetry in response to training in healthy aging (Erickson et al., 2007). Extended cognitive training in MCI may decrease the activations in the compensatory regions and further restore the function of the regions affected by the disease (Kelly et al., 2006b). Figure 1B illustrates a hypothetical model of the interaction between cognitive training, normal aging, and MCI during task performance. Future studies need to test this model in a controlled study.

To examine the effect of cognitive training on brain structure in AD and persons at risk of developing AD, we searched PubMed for the keywords “Alzheimer’s disease” or “MCI” or “Mild Cognitive Impairment”, and “Cognitive Intervention” or “Cognitive Training” or “Cognitive Stimulation”, and “structural MRI” or “VBM” or “FreeSurfer” or “Brain”. We only found one recent study that reported structural changes associated with cognitive training in older adults with subjective memory impairment (SMI; Engvig et al., 2014). Subjects with SMI are at high risk of developing AD (Jessen et al., 2010). The authors reported increased gray matter volume in the left supramarginal and entorhinal regions and in the right inferior temporal and inferior frontal regions after an 8-week memory-training program. The restoration of hippocampal structure after training in SMI group was not significant (Engvig et al., 2014). The limited data suggest a reorganization of memory network (increased cortical volume in the prefrontal cortex) in response to training and favors a compensatory mechanism.

Training-related changes in structural plasticity in persons at risk of developing AD seem to follow the same direction as in healthy aging. However, consistent with our speculation of functional plasticity, it seems that training mainly results in structural plasticity in compensatory regions in at risk subjects while it shows both restorative and compensatory effects in healthy aging. This idea is corroborated by Engvig et al.’s (2014) study that showed restoration of hippocampal structure after training in healthy aging group but not in the SMI group. Extended training in persons at risk of developing AD might result in further restoration of the affected structures.

## Future directions

Recent efforts toward understanding neurocognitive changes associated with cognitive training are promising. However, there are several limitations in the current studies that need to be addressed in the future research. The limitations in the design of the studies, including the lack of active control/placebo group, test-retest reliability of the training outcome, reliability of neuroimaging measures for assessing neuronal change, interaction between the training tasks and outcome measures, compliance, small sample size, lack of effect size information, effects of training on biomarker status (e.g., amyloid deposition), near vs. far transfer and maintenance of gains, have been discussed in previous literature (Clément and Belleville, 2009; van Paasschen et al., 2009; Buschert et al., 2010; Putcha et al., 2011; Belleville and Bherer, 2012; Park and Bischof, 2013). Here we focused on other factors that are critical for understanding the underlying mechanisms of cognitive training.

## Assessing network-level changes associated with cognitive training

In the last decade, there has been increasing evidence regarding aberrant functional and structural connectivity in various stages of AD [see He et al., 2009; Filippi and Agosta, 2011; Radanovic et al., 2013 for a review]. Functional connectivity pattern at rest—specifically the default-mode network connectivity—has gained a lot of attention in recent years as a promising biomarker for tracking changes in AD progression (Koch et al., 2012; Gomez-Ramirez and Wu, 2014). It has been shown that alterations in resting-state network—antedate the pathological effects of amyloid plaque toxicity (Sheline et al., 2010). Reduced integrity in the default mode network is also associated with amyloid beta and tau pathology before the clinical onset of AD (Wang et al., 2013). Several pharmacological intervention studies have employed the resting-state connectivity as a biomarker for testing the efficacy of the treatment (Goveas et al., 2011; Lorenzi et al., 2011; Li et al., 2012) [see Hampel et al., 2011 for a related discussion]. Future cognitive training studies need to adopt a similar approach to test the effect of cognitive training on default-mode network.

With respect to task-related functional connectivity, psychophysiological interactions (PPI) analysis tests task-specific changes in functional connectivity between various brain regions (O’Reilly et al., 2012). Recently, McLaren et al. (2012) proposed the concept of generalized context-dependent PPI (gPPI)—with increased flexibility of statistical modeling—that can reveal complementary information regarding subtle within- and between-network interactions and might be more sensitive compared with resting-state connectivity and task-activation methods. Effective connectivity analysis is an alternative approach that tests the influence of a neural system over another and thus takes into account the directionality of the connections (Friston, 2011). These methods can be employed to investigate the effect of training on brain connectivity during task performance.

Coordinated variations in brain morphology (e.g., volume) among different brain regions have been recently employed to infer large-scale structural covariance networks in health and disease (He et al., 2007; Bernhardt et al., 2011; Hosseini and Kesler, 2013; Hosseini et al., 2013; Singh et al., 2013). The co-occurring atrophy in regional volume in the default-mode network and medial temporal structures has been shown to relate to elevated levels of amyloid beta and tau pathology (Carmichael et al., 2012). Other studies reported alterations in the global and regional organization of structural covariance networks associated with MCI and AD (He et al., 2008; Yao et al., 2010). These networks can be used to identify the long-term effects of training on brain networks.

Despite the clear network-level substrate for cognitive deficits in AD, there is only one study that investigated changes in brain connectivity in response to memory training in MCI patients (Hampstead et al., 2011). Using granger causality analysis, Hampstead et al. (2011) found increased effective connectivity in the middle temporal gyrus, precuneus and occipital cortex after training. These findings could help the authors clarify how explicit memory training provides a mechanism to recruit compensatory memory processes mediated by the posterior default mode network. Future studies need to investigate the effects of training on brain connectivity and organization of brain networks in order to dissociate the compensatory and restorative effects of training at network level.

### Individual differences in response to training

While causes of AD are yet to be understood, there is an agreement that AD develops as a result of multiple risk factors. Genetic, demographic and host factors play an important role in AD development. Presence of APOE-ɛ4 allele has been associated with increased rate of cognitive decline and increased neuropathology including greater β-amyloid deposition and medial temporal lobe atrophy (Ohm et al., 1995; Dal Forno et al., 1996; Geroldi et al., 1999; Martins et al., 2005; Lim et al., 2013). Higher educational attainment has also been associated with reduced risk of AD in multiple studies (McDowell et al., 2007; Roe et al., 2007; Meng and D’Arcy, 2012) potentially through cognitive reserve mechanism (Stern, 2006; Brayne et al., 2010). The cognitive reserve theory posits that individual differences in brain structure and/or efficiency to information processing provide differential protection against brain pathology or age-related changes (Stern, 2006). Higher educational attainment can thus delay the onset of dementia through mitigating the impact of pathology on the clinical expression of AD (Brayne et al., 2010).

Disease stage and individual differences in the pattern of pathology would also impact the training outcomes. Unfortunately, little information is available on the effect of individual differences on training outcomes. A recent study has tested the effect of individual’s hippocampal subfield volume on memory training outcomes in SMI and reported that subjects with larger hippocampal volume at baseline would gain greater improvement in verbal recall after training (Engvig et al., 2012b). Integrative studies need to address the effect of individual differences in genetic and demographic factors on training outcomes that further helps to design efficient cognitive training programs. Specifically, multivariate pattern analysis can be employed to predict with high accuracy how an individual would respond to training using the neuroimaging, genetic and demographic information at baseline. These techniques have been extensively used in neuroimaging literature for predicting disease and/or disease stage (Bray et al., 2009; Hoeft et al., 2011; Orrù et al., 2012). Finally, neuroimaging measures can be employed to identify the brain regions (networks) that are affected by the disease at individual level. In recent years, there has been an increase in the number of studies that compare the single case data against a control group (Rosen et al., 2002; Zahn et al., 2005; Sehm et al., 2011). Although the results of such single-case studies are still controversial (Scarpazza et al., 2013), these studies can be quite helpful to identify the target network for cognitive training and to investigate how the function/structure of those networks are recovered after training. Neurofeedback technology can also be employed to target the identified networks (Koush et al., 2013). These technologies will ultimately lead to customizing cognitive training programs for individuals.

### Multi-domain cognitive training

While most of the neuroimaging studies of cognitive training in AD have focused on memory training, the results suggest an increased activity and gray matter volume in various prefrontal regions (Figure 1A). Previous neuroimaging studies have indicated the involvement of prefrontal regions in memory formation and suggest that the likelihood of memory formation correlates with the level of activity in prefrontal regions (Buckner et al., 1999; Peleg-Raibstein et al., 2005). Executive functions (EFs), specifically the working memory components, have been shown to exacerbate memory deficits and could represent a critical factor in AD progression (Ranganath et al., 2005; Nagata et al., 2011; Parks et al., 2011; Clément et al., 2013). It has been shown that EF in MCI patients, and not in normal aging, declines faster than memory (Johnson et al., 2012). Functional neuroimaging studies on MCI showed hyper-activation and hypo-activation in prefrontal regions in MCI patients with high and low cognitive functions, respectively (Dannhauser et al., 2005; Yetkin et al., 2006), an observation that suggest a breakdown of executive function network with progression of AD. These data are corroborated by recent evidence that showed impairments in EF even in early stages of AD (Rainville et al., 2012; Clément et al., 2013). These data suggest that including training components focused on EF in the practicum is necessary to restore/normalize the affected structure/function of the prefrontal regions and to further facilitate the recovery of the medial temporal regions. This idea is supported by a recent study that provided behavioral evidence of improvement in memory processes as a result of working memory training (Rudebeck et al., 2012).

Multi-domain cognitive training programs have the potential to maximize the restorative/normalizing effects of training that is the ideal goal of training. Gates et al. reviewed the behavioral effects of cognitive training on normal aging, MCI and dementia and suggested that multi-domain cognitive training has the potential to improve cognitive functions in healthy older adults and slow the cognitive decline in MCI population (Gates and Valenzuela, 2010). Compared with single-domain training, multi-domain cognitive training is more advantageous in terms of training-effect maintenance in non-demented older adults (Cheng et al., 2012). Additionally, parallel practice is believed to improve transfer effects of training to untrained and/or real-world skills (Green and Bavelier, 2008). The effect of multi-domain cognitive training on brain structure and function in AD needs to be investigated in the future studies.

In summary, we reviewed the neuroimaging studies that investigated the effects of cognitive training on brain structure and function in persons at risk of developing AD. Current data suggest that brain structural and functional plasticity advance into old age and that cognitive training can be employed to restore neurocognitive decline associated with AD. Training in persons at risk of developing AD was associated with increased brain activity and gray matter volume across studies that mainly indicate recruitment of compensatory mechanisms. Future multi-modal neuroimaging studies with focus on training various cognitive domains need to investigate the effect of training on the diffuse pathology associated with AD. Additionally, future neuroimaging research need to examine the individual differences in response to training and clarify the role of genetic and host factors on training outcome.

## Conflict of interest statement

The authors declare that the research was conducted in the absence of any commercial or financial relationships that could be construed as a potential conflict of interest.
